# 한국 비결핵항산균 폐질환 환자의 건강관련 삶의 질 영향요인: 횡단적 조사 연구

**DOI:** 10.7586/jkbns.25.042

**Published:** 2025-11-21

**Authors:** Seeun Jeon, Yoonju Lee

**Affiliations:** 1 Pusan National University Hospital, Busan, Korea; 1 부산대학교병원; 2 College of Nursing・Research Institute of Nursing Science, Pusan National University, Yangsan, Korea; 2 부산대학교 간호대학・간호과학연구소

**Keywords:** Nontuberculous mycobacteria, Quality of life, Signs and symptoms, Uncertainty, Health behavior, 비결핵항산균, 삶의 질, 징후 및 증상, 불확실성, 건강행위

## Abstract

**Purpose:**

This study aimed to identify the factors influencing health-related quality of life (HRQoL) in Korean patients with non-tuberculous mycobacterial pulmonary disease (NTM-PD).

**Methods:**

A cross-sectional study was conducted with 130 outpatients diagnosed with NTM-PD at Pusan National University Hospital between August and November 2023. Data were obtained using validated questionnaires assessing symptom experience, illness uncertainty, health-promoting behaviors, and HRQoL. Clinical characteristics were extracted from medical records. Statistical analyses included descriptive statistics, t-tests, analysis of variance, Pearson correlation coefficients, and multiple regression analysis performed with SPSS version 27.0.

**Results:**

The mean physical and mental HRQoL scores were 46.82 ± 6.33 and 43.95 ± 11.42, respectively, both significantly lower than the population norm of 50. Physical HRQoL was negatively associated with symptom experience (B = −0.48, *p* < .001) and illness uncertainty (B = −0.08, *p* = .004), with these variables explaining 40%–53% of the variance. Mental HRQoL was adversely affected by symptom experience (B = −0.89, *p* < .001) and illness uncertainty (B = −0.15, *p* = .001), while positively associated with health-promoting behaviors (B = 0.15, *p* = .001), family cohabitation (B = 4.93, *p* = .045), and obesity status (B = 9.24, *p* = .004). Together, these factors explained 53–55% of the variance in mental HRQoL.

**Conclusion:**

HRQoL in patients with NTM-PD is significantly impaired and influenced by multiple factors. Evidence-based nursing interventions should emphasize symptom management, reduction of illness uncertainty, promotion of health-promoting behaviors, and strengthening of family support systems to optimize patient outcomes and overall well-being.

## 서론

### 1. 연구의 필요성

비결핵항산균(non-tuberculous mycobacteria, NTM)은 결핵균과 나병균을 제외한 항산균으로, 주로 토양과 물 등 자연환경에 존재하며 인체에 감염을 유발할 수 있다[1]. 현재까지 190종이 넘는 균주가 알려져 있는데[2] 이 중 일부는 인체에 감염되어 폐질환, 림프절염, 피부 감염 등을 유발하며, 이 중 폐질환은 NTM 감염 중 가장 흔한 형태로 전체 감염의 90% 이상을 차지한다[1]. 비결핵항산균 폐질환(non-tuberculous mycobacterial pulmonary disease, NTM-PD)은 치료가 어렵고 재발률이 높은 만성 호흡기질환으로 최근 국내외에서 이 질환의 유병률이 급증하고 있다[3]. 국내의 경우 2010년 인구 10만 명당 11.4건에서 2021년 56.7건으로 약 5배로 증가하였으며, 같은 기간 결핵 유병률은 감소하여 2021년에는 NTM-PD의 유병률이 결핵을 넘어서는 역전 현상이 나타났다[4].

NTM-PD는 완치를 보장하기 어려운 최소 1년 이상의 장기 치료가 필요하며, 복합 항생제 치료로 인해 심각한 부작용이 동반되기도 한다[2]. 치료 성공률은 약 60%에 그치며, 치료 종료 후 30%가 재발하는 것으로 보고되어[5,6] 단순한 미생물학적 완치만으로는 환자의 전반적인 회복을 기대하기 어렵다. 또한 NTM-PD 환자는 긴 치료 과정에서 신체적 증상, 부정적인 감정, 치료에 대한 부담 등으로 삶의 질이 저하되므로[7] NTM-PD 환자의 건강관련 삶의 질을 유지하고 향상하는 것이 중요한 치료 목표로 대두되고 있다[8].

건강관련 삶의 질이란 질병이나 치료에 의해 영향을 받는 삶의 신체적, 심리적, 사회적 측면을 개인이 주관적으로 평가하는 건강 상태를 의미한다[9]. NTM-PD 환자는 일반 인구보다 건강관련 삶의 질이 현저히 낮아 신체적 활동 및 사회적 활동에 장애가 발생한 것으로 나타났다[10]. 또한 NTM-PD 환자의 건강관련 삶의 질은 폐결핵 환자의 삶의 질과 유사하게 낮은 수준으로 신체적, 정신적 삶의 질이 저하된 것으로 나타났다[11,12]. 국내외 NTM-PD 환자를 대상으로 한 선행연구에서 연령, 흡연 등의 인구사회학적 요인과 C 반응성 단백(C-reactive protein, CRP), 폐기능 상태, 항산균 도말검사 결과 등의 임상적 요인이 NTM-PD 환자의 삶의 질에 영향을 주는 것으로 보고되었다[11,13].

NTM-PD 환자들은 만성 기침, 객담, 피로, 호흡곤란 등의 증상을 지속적으로 경험한다[1]. 증상경험이란 정상 기능의 변화에 대한 개인의 지각, 증상의 의미에 대한 평가로 신체적, 심리적, 사회적, 행동 구성요소를 포함하는 증상에 대한 반응을 의미한다[14]. NTM-PD의 증상은 NTM-PD의 진단과 치료를 시작하는 주요 기준이 되고[2], 치료에 대한 반응의 중요한 지표가 된다[15]. NTM-PD 환자들의 증상 발현은 무증상부터 중등도 이상까지 다양한데[16], 증상경험이 심할수록 단순한 생리적 불편함을 넘어 신체적 활동 및 사회적 참여에 제한을 받고, 삶의 질에 부정적인 영향을 미치는 것으로 나타났다[17]. 특히 만성 객담이 동반된 환자들은 폐기능 저하와 심각한 방사선학적 소견을 보이는 경우가 많으며, 그렇지 않은 환자에 비해 삶의 질이 더욱 낮은 것으로 보고되었다[11]. 증상에 대한 경험 정도가 많을수록 자신에 대한 건강 수준 및 삶의 질을 부정적으로 인식하므로[17] 삶의 질 향상을 위해서는 질병 과정에서 겪는 증상 완화에 대한 접근이 중요하다.

NTM-PD 감염의 임상 증상은 비특이적이고[15], NTM-PD의 진단은 배양검사에서 최종 결과를 얻기까지 최소 8주 이상의 오랜 시간이 필요하다[1]. 또한 진단 기준을 충족하더라도 반드시 항생제 치료가 필요하지는 않으며[2], 치료를 시작하더라도 다양한 부작용 등으로 인해서 제한적이다[15]. 이러한 질병의 진단과 치료 과정에서 모호함, 예측할 수 없는 질병의 경과, 정확한 정보 부족으로 인해 NTM-PD 환자는 불확실성을 경험하게 된다[18]. 불확실성이란 친숙하지 않은 질병과 관련된 사건이나 예측 불가능한 증상, 질병의 경과 및 예후에 대한 불충분한 정보, 확신할 수 없는 치료 결과로 발생할 수 있는 인지적 상태를 말한다[19]. NTM-PD와 같이 유병 기간이 긴 만성질환자에서 불확실성이 높을수록 질병에 대한 대처가 낮아지며 건강관련 삶의 질이 저하된 것으로 보고되었다[20]. NTM-PD 환자는 장기간 반복되는 악화, 입원, 장기 약물치료, 예후에 대한 불확실성, 그리고 치료 실패의 가능성 등으로 인해 심리적 부담과 삶의 질 저하를 경험하고[7], 특히 질병의 예측 불가능성은 환자와 가족 모두에게 스트레스를 준다[21]. 이에 NTM-PD 환자에서 불확실성이 건강관련 삶의 질에 부정적인 영향을 미칠 것으로 예측되므로 그 관계를 확인할 필요가 있다.

이러한 상황에서 NTM-PD 환자의 삶의 질을 향상하기 위해서는 질병 관리에 대한 개인의 적극적인 노력, 즉 건강증진행위가 중요하다[22]. NTM-PD는 물, 토양 등의 환경적 요인과 식습관, 저체중, 흡연 등의 건강관련 생활 습관 요인들이 위험인자로 밝혀졌다[23]. 또한 기관지확장증, 폐결핵 등의 만성폐질환 환자나 인간 면역결핍 바이러스(human immunodeficiency virus) 환자 등의 면역저하자에서 동반 이환되어 쉽게 발생하는 것으로 나타났다[23]. NTM-PD의 치료 완치율을 높이고 재발률을 낮추기 위해서는 끓인 물을 섭취하고 마스크를 착용하는 등의 물과 토양에서 NTM의 노출을 줄이는 생활 방식의 개선과[24] 체중 유지 및 영양 섭취를 통한 면역관리가 필요하다[18]. 따라서 NTM-PD 환자의 건강관련 삶의 질을 향상하기 위해서 그들의 건강증진행위의 수준을 확인하고 건강관련 삶의 질과의 관계를 파악할 필요가 있다.

NTM-PD에 관한 연구가 증가하고 있지만 현재까지의 연구는 주로 질병의 진단[1,15]과 치료[2,5]에 관한 내용에 집중되어 NTM-PD 환자의 건강관련 삶의 질에 영향을 미치는 요인을 파악하기에는 제한적이다. 이에 본 연구는 NTM-PD 환자를 대상으로 인구사회학적 및 질병 관련 특성, 증상경험, 불확실성, 건강증진행위가 건강관련 삶의 질에 미치는 영향을 파악하고자 한다.

### 2. 연구의 목적

본 연구의 목적은 NTM-PD 환자의 건강관련 삶의 질에 영향을 미치는 요인을 규명하기 위함이며, 구체적인 목적은 다음과 같다.

1) 대상자의 특성과 특성에 따른 건강관련 삶의 질 차이를 파악한다.

2) 대상자의 증상경험, 불확실성, 건강증진행위 및 건강관련 삶의 질 정도를 파악한다.

3) 대상자의 증상경험, 불확실성, 건강증진행위 및 건강관련 삶의 질 간의 상관관계를 파악한다.

4) 대상자의 건강관련 삶의 질에 미치는 영향요인을 규명한다.

## 연구 방법

### 1. 연구 설계

본 연구는 NTM-PD 환자의 건강관련 삶의 질의 정도와 이에 영향을 미치는 요인을 규명하기 위한 서술적 조사연구이다.

### 2. 연구 대상

본 연구의 대상자는 부산시에 소재하는 상급종합병원인 부산대학교병원 호흡기내과에서 NTM-PD를 진단받고 외래에서 추적관찰 중인 환자를 대상으로 하였다. 단, 인지기능에 문제가 있는 자, 항암화학요법이나 방사선요법 등의 치료를 받는 자, 우울증을 진단받고 치료를 받는 자는 제외하였다. 표본 수는 G*Power 3.1.9.7 프로그램을 이용하여 다중회귀분석에 필요한 표본 수를 산정하였다[25]. 표본 수 산출을 위해 NTM-PD와 유사한 폐결핵 환자의 삶의 질 영향요인을 분석한 선행연구[26]를 참고하여 효과크기 .35, 유의수준 .05, 검정력 .95, 예측변수 22개를 기준으로 다중회귀분석에 필요한 최소 대상자 수는 총 109명이었다. 탈락률 20%를 고려하여 총 137명의 자료를 수집하였으며, 주요 변수의 응답이 누락된 7부를 제외하여 총 130부를 최종 분석에 사용하였다.

### 3. 연구 도구

본 연구에서 사용된 자가보고형 설문은 대상자의 인구사회학적 특성, 증상경험, 불확실성, 건강증진행위, 건강관련 삶의 질로 구성하였다. 대상자의 임상적 특성은 연구자가 의무기록을 검토하여 조사하였다.

#### 1) 대상자의 특성

대상자의 특성 변수는 NTM-PD 환자 및 폐결핵 환자를 대상으로 삶의 질을 분석한 선행연구[11,12]를 참고하여 구성하였다. 대상자의 인구사회학적 특성은 성별, 연령, 결혼 상태, 동거가족 유무, 최종 학력, 직업 유무, 흡연 상태를 포함하였다. 대상자의 임상적 특성은 균종, 항산균 도말검사 결과, CT 상 공동 유무, CRP, 적혈구 침강 속도(erythrocyte sedimentation rate, ESR), 유병 기간, 항생제 치료 경험, 입원 치료 경험 유무, 과거 폐질환 유무, 체질량지수(body mass index, BMI)를 포함하였다. BMI는 체중(kg)을 키의 제곱(m^2^)으로 나눈 값을 의미하며, 저체중(18.5 kg/m^2^ 미만), 정상 체중(18.5∼22.9 kg/m^2^), 과체중(23∼24.9 kg/m^2^), 비만(25 kg/m^2^ 이상)으로 분류한다[27]. 임상 및 역학 연구에서 생체지표의 유효성과 자료의 일관성을 확보하기 위해 일반적으로 6개월을 기준으로 하며, NTM-PD 가이드라인에서 대부분의 *Mycobacterium Avium* Complex (MAC) 환자들이 치료 시작 후 6개월 이내에 균 전환을 보이는 것으로 보고된다[2]. 이에 모든 검사 결과는 자가 보고형 설문을 작성한 날을 기준으로 이전 6개월 이내에 시행된 결과 중 가장 최근의 결과를 조사하였다.

#### 2) 증상경험

증상경험은 Park [28]이 개발한 만성폐쇄성폐질환자의 신체적 증상경험 측정도구를 Song 등[29]이 폐결핵 환자에게 맞게 수정·보완한 도구를 저자의 승인을 받아 사용하였다. 질병관리청의 결핵진료지침[30]과 선행연구[1]를 검토한 결과, 폐결핵과 NTM-PD는 기침, 피로, 발열, 야간발한, 식욕부진 등의 주요 증상이 유사하게 나타나는 것으로 확인되었다. 따라서 폐결핵 환자를 대상으로 개발된 증상경험 도구를 NTM-PD 환자에게 적용하는 것이 타당하다고 판단하였다. 도구는 기침, 가래, 호흡곤란, 피곤, 식욕부진 등의 신체 증상을 측정하는 16개 문항으로 구성된다. 각 문항은 ‘경험 없다(1점)’, ‘가끔 그렇다(2점)’, ‘자주 그렇다(3점)’, ‘항상 그렇다(4점)’으로 측정하며, 총점이 높을수록 증상경험이 심함을 의미한다. Song 등[29]의 연구에서 Cronbach’s α는 .85이었고, 본 연구에서는 .74이었다.

#### 3) 불확실성

불확실성은 Mishel [19]이 개발한 질병의 불확실성 척도(Mishel’s Uncertainty in Illness Scale)를 Chung 등[31]이 번역하여 수정한 도구를 저자의 승인을 받아 사용하였다. 도구는 모호성(13문항), 복합성(7문항), 불일치성(7문항), 불예측성(5문항)의 4개 하위영역과 미분류 1문항으로 구성되어 있다. 각 문항은 ‘전혀 아니다(1점)’, ‘아니다(2점)’, ‘그저 그렇다(3점)’, ‘그렇다(4점)’, ‘매우 그렇다(5점)’로 측정하며, 점수가 높을수록 불확실성이 높음을 의미한다. Chung 등[31]의 연구에서 Cronbach’s α는 .85이었고, 본 연구에서는 .92이었다.

#### 4) 건강증진행위

본 연구에서는 Walker 등[32]이 개발한 Health Promoting Lifestyle Profile을 Byeon과 Hyun [33]이 진폐 환자를 대상으로 수정·보완한 도구를 저자의 승인을 받은 후, ‘진폐’를 ‘비결핵항산균 폐질환’으로 수정하여 사용하였다. 본 도구는 영양(6문항), 운동(4문항), 휴식 및 스트레스(8문항), 대인관계(6문항), 건강관리(10문항), 자아실현(6문항)의 6개 하위영역, 총 40개 문항으로 구성되어 있다. 각 문항은 ‘전혀 그렇지 않다(1점)’, ‘대체로 그렇지 않다(2점)’, ‘보통 그렇다(3점)’, ‘대체로 그렇다(4점)’, ‘매우 그렇다(5점)’로 측정하며, 점수가 높을수록 건강증진행위를 많이 함을 의미한다. Byeon과 Hyun [33]의 연구에서 Cronbach’s α는 .89이었고, 본 연구에서는 .94이었다.

#### 5) 건강관련 삶의 질

본 연구에서는 Ware 등[34]이 개발한 The Short Form-36 Health Survey version 2 (SF-36v2)의 한국어판을 저작권자인 QualityMetric Inc.로부터 제공받아 사용하였다. SF-36v2는 건강관련 삶의 질을 평가하기 위한 자가보고형 도구로 총 36개 문항으로 구성되어 있으며, 신체적 삶의 질과 정신적 삶의 질 두 영역으로 구분하여 평가한다. 신체적 삶의 질은 신체적 기능(10문항), 신체적 역할 제한(4문항), 통증(2문항), 전반적인 건강상태(5문항)로 구성되고, 정신적 삶의 질은 활력(4문항), 사회적 기능(2문항), 정서적 역할 제한(3문항), 정신 건강(5문항)으로 구성되며, 각 문항은 3점, 5점, 6점 Likert 척도가 혼합 구성되어 있다. 수집된 자료는 QualityMetric Inc.에서 제공하는 점수산출 프로그램을 이용하여 분석하여 미국 일반 인구를 기준으로 평균 50, 표준편차 10이 되도록 선형 변환하는 Norm-based scoring (NBS) 점수를 산출하였으며, 점수가 높을수록 건강관련 삶의 질이 높음을 의미한다. Ware 등[34]의 연구에서 Cronbach’s α는 신체적 삶의 질 .95, 정신적 삶의 질 .93이었고, 본 연구에서는 신체적 삶의 질 .90, 정신적 삶의 질 .94이었다.

### 4. 자료 수집

본 연구의 자료 수집은 부산광역시에 소재한 부산대학교병원의 호흡기내과에서 NTM-PD를 진단받고 외래에서 추적관찰 중인 환자를 대상으로 2023년 8월 11일부터 2023년 11월 27일까지 이루어졌다. 연구 시행 전 해당 병원의 간호부와 호흡기내과 교수에게 연구 목적을 설명하여 진행 허가를 받았으며, 보건의료정보팀에 전자의무기록 열람 승인을 받은 후 진행하였다. 연구자가 직접 대상자에게 연구의 목적과 방법을 설명하고 서면 동의한 대상자에게 구조화된 설문지를 배부하였고, 별도로 마련된 회수함을 설치하여 회수하였다. 연구 대상자가 스스로 설문지를 작성하는 것을 원칙으로 하였으며, 시력 저하 등의 신체적 제약으로 독립적인 작성이 어려운 경우에만 연구보조자가 설문 문항을 읽어주고 응답을 대신 기록하였다. 이때 응답의 객관성을 확보하기 위해 문항 내용 외의 부가적인 설명이나 유도는 하지 않았다. 설문지 작성은 20분 정도 소요되었고, 연구자는 설문을 완료한 대상자의 전자의무기록을 열람하여 대상자의 임상적 특성 자료를 수집하였다.

### 5. 자료 분석

본 연구에서는 수집된 데이터의 정규성을 검증하기 위해 Kolmogorov-Smirnov 검정, Shapiro-Wilk 검정을 실시하고, 왜도와 첨도 값을 확인하였으며, Q-Q plot을 통한 시각적 검토를 병행하였다. 정규성 검정 결과 일부 변수에서 유의확률이 *p* < .05로 나타나 정규분포 가정을 충족하지 않는 것으로 확인되었다. 그러나 모든 측정변수의 왜도와 첨도 절댓값이 2.0 미만으로 나타났으며, Q-Q plot 분석 결과 대부분의 데이터점이 대각선 근처에 분포하여 심각한 정규성 위배는 없는 것으로 판단하여 모수통계 분석을 실시하였다. 수집된 자료는 SPSS version 27.0 (IBM Corp., Armonk, NY, USA) 프로그램을 이용하여 분석하였으며, 모든 통계적 유의성은 *p* < .05를 기준으로 하였다. 구체적인 자료 분석 방법은 다음과 같다.

1) 대상자의 특성, 증상경험, 불확실성, 건강증진행위 및 건강관련 삶의 질은 기술통계로 분석하였다.

2) 대상자의 특성에 따른 건강관련 삶의 질의 차이는 t-test와 one-way ANOVA를 이용하여 분석하였고, 사후검증은 Scheffé test를 이용하여 검증하였다.

3) 대상자의 증상경험, 불확실성, 건강증진행위 및 건강관련 삶의 질의 상관관계는 Pearson correlation coefficient로 분석하였다.

4) 대상자의 건강관련 삶의 질에 영향을 미치는 요인을 규명하기 위하여 다중회귀분석을 수행하였다. 결측값 처리(ESR, CRP)를 위해 다중대체법을 적용하였는데, 총 5개의 대체 데이터 세트를 생성하여 각각 회귀분석을 수행한 후 Rubin's Rules [35]을 이용하여 통합한 결과를 사용하였다.

### 6. 윤리적 고려

본 연구는 부산대학교의 생명윤리위원회(Institutional Review Board, IRB)의 연구 승인(PNU IRB/2023_84_HR)을 받은 후 진행하였다. 연구자가 대상자에게 본 연구의 목적과 절차, 익명성과 비밀 보장에 관한 내용, 연구 참여를 원하지 않는 경우 언제든지 중단할 수 있으며 중도 포기로 인한 어떠한 불이익도 없음을 설명한 후 자발적으로 연구 참여에 동의한 경우 서면 동의서를 받고 자료수집을 진행하였다. 수집된 자료에서 대상자의 개인식별정보(환자 등록번호)는 개인정보 보호법에 따라 일련번호를 부여하여 익명화하여 사용하였고, 연구자료는 연구 종료 후 3년간 보관하며 이후에는 완전히 폐기될 것임을 설명하였다. 모든 연구 참여자에게는 설문이 완료된 후 소정의 답례품을 제공하였다.

## 연구 결과

### 1. 대상자의 특성에 따른 제변수간 차이

대상자의 인구사회학적 특성에서 성별은 여성이 97명(74.6%)으로 많았으며, 평균 연령은 65.56 ± 8.97세로 65세 이상이 71명(54.6%)을 차지했다. 결혼 상태는 기혼자가 101명(77.7%)이었고, 가족과 동거하는 경우가 109명(83.8%)이었으며, 직업이 없는 대상자가 82명(63.1%)이었다. 임상적 특성을 살펴보면, NTM의 균종은 MAC가 80명(61.5%)으로 가장 높은 비율을 보였고, 항산균 도말검사 양성자는 41명(31.5%)이었다. CRP 수치가 0.5 mg/dL을 초과한 대상자가 17명(20.2%), ESR 수치가 20 mm/hr을 초과한 대상자가 47명(51.6%)이었다. 질병 유병 기간이 평균 42.61 ± 27.70개월이었으며, 37∼60개월 구간이 38명(29.2%)으로 가장 많았다. 현재 항생제 치료 중인 대상자가 65명(50.0%)이었고, 입원 치료 경험이 있는 대상자는 49명(37.7%)이었다. BMI는 정상 체중군이 78명(60.0%)으로 가장 높은 비율을 차지했다(Table 1).

대상자의 특성에 따른 건강관련 삶의 질 차이를 분석한 결과는 다음과 같다. 신체적 삶의 질은 성별(t = 2.32, *p* = .024), 결혼 상태(t = 2.08, *p* = .044), 동거가족 유무(t = 2.13, *p* = .042), 직업 유무(t = 2.64, *p* = .010) 등 인구사회학적 특성과 CRP 수치(t = 4.51, *p* < .001), ESR 수치(t = 3.17, *p* = .002), 입원 치료 경험(t = −2.01, *p* = .048) 등 임상적 특성에 따라 유의한 차이를 보였다. 정신적 삶의 질은 결혼 상태(t = 1.97, *p* = .041), 동거가족 유무(t = 2.39, *p* = .023), 직업 유무(t = 2.02, *p* = .046) 등 인구사회학적 특성과 CRP 수치(t = 3.50, *p* = .002), ESR 수치(t = 2.10, *p* = .038), 입원 치료 경험(t = −2.33, *p* = .022), 과거 폐질환 유무(t = −2.19, *p* = .036) 등 임상적 특성에 따라 유의한 차이가 나타났다. 또한 BMI가 높은 군일수록 정신적 삶의 질이 높은 경향을 보였으나(F = 3.40, *p* = .020), 사후분석 결과 개별 집단 간 통계적 유의성은 확인되지 않았다(Table 1).

### 2. 대상자의 증상경험, 불확실성, 건강증진행위, 건강관련 삶의 질

대상자의 증상경험 총점은 64점 만점에 평균 25.91 ± 4.92점, 불확실성 총점은 160점 만점에 평균 93.96 ± 18.08점, 건강증진행위 총점은 200점 만점에 평균 142.87 ± 20.08점이었다. 신체적 삶의 질은 46.82 ± 6.33점, 정신적 삶의 질은 43.95 ± 11.42점으로 나타났다(Table 2).

### 3. 대상자의 증상경험, 불확실성, 건강증진행위, 건강관련 삶의 질 간의 상관관계

대상자의 신체적 삶의 질은 증상경험(r = −.57, *p* < .001), 불확실성(r = −.43, *p* < .001)과 음의 상관관계가 있고, 건강증진행위(r = .39, *p* < .001)와는 양의 상관관계가 있었다. 정신적 삶의 질 또한 증상경험(r = −.61, *p* < .001), 불확실성(r = −.48, *p* < .001)과 음의 상관관계가 있었고, 건강증진행위(r = .56, *p* < .001)와는 양의 상관관계가 있었다(Table 3).

### 4. 대상자의 건강관련 삶의 질 영향요인

본 연구에서는 건강관련 삶의 질에 영향을 미치는 요인을 파악하기 위하여 검사 결과가 없는 CRP와 ESR에 대하여 다중 대체법(multiple imputation)을 사용하여 다중회귀분석을 실시하였다. 다중 대체 데이터 세트는 5개를 사용하였으며, 다중회귀분석 결과는 5개의 결과를 범위로 제시하였고, 최종 회귀계수와 유의성 검정은 Rubin's Rules [35] 에 따라 통합된 결과를 제시하였다.

신체적 삶의 질 영향요인을 파악하기 위해 단변량 분석에서 통계적으로 유의한 변수인 성별, 결혼 상태, 동거가족 유무, 직업 유무, CRP, ESR, 입원 치료 경험 유무, 증상경험, 불확실성, 건강증진행위를 독립변수로 투입하였으며, 성별, 결혼 상태, 동거가족 유무, 직업 유무, CRP, ESR, 입원 치료 경험 유무는 가변수(dummy variable)로 처리하였다. 다중회귀분석을 위한 가정을 검증한 결과, Durbin-Watson 값은 1.64∼1.79로 2에 근사한 값을 보여 오차의 자기상관이 없음을 확인하였다. 잔차의 정규성은 각 데이터 세트별 히스토그램 및 정규확률 P-P Plot을 통해 검토한 결과, 잔차가 대체로 직선 형태를 따르는 것으로 나타나 정규성 가정을 충족하였다. 잔차 산점도를 분석한 결과 예측값과 잔차 간에 일정한 분포를 보여 등분산성 가정도 만족하는 것으로 확인되었다. 다중공선성 검토 결과, 모든 세트에서 독립 변수의 공차한계(tolerance)가 0.49∼0.91, 분산팽창인자(variance inflation factor)는 1.10∼2.04로 나타나 다중공선성에 문제가 없는 것으로 확인되었다. 회귀 모형의 설명력은 수정된 R^2^ 기준으로 .40∼.53이었으며, 모든 모형에서 통계적으로 유의하였다(*p* < .001). 다중회귀분석 결과 증상 경험이 많을수록(B = −0.48, *p* < .001), 불확실성이 클수록(B = −0.08, *p* = .004) 신체적 삶의 질이 감소하는 것으로 나타났다(Table 4).

다음으로 정신적 삶의 질 영향요인을 파악하기 위해 단변량 분석에서 통계적으로 유의한 변수인 결혼 상태, 동거가족 유무, 직업 유무, CRP, ESR, 입원 치료 경험 유무, BMI, 증상경험, 불확실성, 건강증진행위를 독립변수로 투입하였으며, 결혼 상태, 동거가족 유무, 직업 유무, CRP, ESR, 입원 치료 경험 유무, BMI는 가변수로 처리하였다. 다중회귀분석을 위한 가정을 검증한 결과, Durbin-Watson 값은 1.78∼1.89로 2에 근사한 값을 보여 오차의 자기상관이 없음을 확인하였다. 잔차의 정규성은 각 데이터 세트별 히스토그램 및 정규확률 P-P Plot을 통해 시각적으로 검토한 결과, 잔차가 대체로 직선 형태를 따르는 것으로 나타나 정규성 가정을 충족하였다. 잔차 산점도를 분석한 결과 예측값과 잔차 간에 일정한 분포를 보여 등분산성 가정도 만족하는 것으로 확인되었다. 다중공선성 검토 결과, 모든 세트에서 독립변수의 공차한계가 0.47∼0.93, 분산팽창인자는 1.08∼2.12로 나타나 다중공선성에 문제가 없는 것으로 확인되었다. 회귀 모형의 설명력은 수정된 R^2^ 기준으로 .53∼.55이었으며, 모든 모형에서 통계적으로 유의하였다(*p* < .001). 다중회귀분석 결과 증상 경험이 많을수록(B = −0.89, *p* < .001), 불확실성이 클수록(B = −0.15, *p* = .001) 정신적 삶의 질이 낮았고, 건강증진행위를 많이 할수록(B = 0.15, *p* = .001), 가족과 함께 거주하거나(B = 4.93, *p* = .045) BMI가 비만군에 해당하는 경우(B = 9.24, *p* = .004) 정신적 삶의 질이 높게 나타났다(Table 5).

## 논의

본 연구는 NTM-PD 환자의 건강관련 삶의 질 수준을 파악하고 건강관련 삶의 질에 영향을 미치는 요인을 규명하고자 수행되었다. 본 연구의 주요 결과를 중심으로 다음과 같이 논의하고자 한다.

본 연구 대상자의 인구사회학적 특성을 살펴보면, 여성이 74.6%로 남성보다 현저히 높게 나타났다. 이는 Yamane 등[11]의 94.9%, Kwak 등[36]의 72.8%와 일치하는 결과로, NTM-PD에서 관찰되는 일관된 성별 분포 특성을 확인해준다. 이러한 여성 편중 현상은 중년 이후 여성의 경우 가사 노동 중 환경 노출 기회 증가, 호르몬 변화로 인한 면역력 저하 등이 복합적으로 작용한 결과로 해석된다. 평균 연령은 65.56세로 고령층의 비율이 높았으며, 이는 일본에서 시행된 선행연구[11]의 평균 연령 67세와 유사한 결과이다. 이를 통해 NTM-PD가 고령층에서 호발하는 질환임을 보여준다. 임상적 특성에서 평균 유병 기간은 42.61개월이었으며, 유병 기간이 12개월을 초과한 대상자가 86.2%, 항생제 치료 경험이 있는 대상자가 89.2%에 달했다. 이러한 결과는 NTM-PD가 장기적인 추적관찰과 치료가 필요한 만성질환임을 보여준다. 실제로 임상 지침에서는 증상이 경미하거나 방사선학적 소견이 뚜렷하지 않은 환자에게도 질병 진행 위험을 고려하여 정기적인 장기 추적관찰을 권고하고 있으며[18], 완치를 위해서는 수개월에서 수년간의 다제 항생제 치료가 필요하다[2]. 이와 같은 장기간에 걸친 치료 과정은 환자에게 상당한 부담을 초래하고, 지속적인 약물 복용과 치료 과정에서 발생하는 부작용은 환자의 신체적 고통뿐만 아니라 정서적 피로감을 유발하여 전반적인 삶의 질을 저하시키는 주요 요인이 된다[37]. 따라서 NTM-PD 환자가 장기 치료 과정에서 경험하는 불안과 스트레스를 완화하고 효과적인 자기 관리를 지원하여 궁극적으로 건강관련 삶의 질을 향상시킬 수 있는 통합적 접근 방안이 필요하다.

본 연구에서는 건강관련 삶의 질 측정을 위해 SF-36v2를 사용하였으며, 미국 일반 인구를 기준으로 평균 50점, 표준편차 10점이 되도록 선형 변환하는 NBS 점수를 적용하였다[34]. 연구 결과 대상자의 신체적 삶의 질은 46.8점, 정신적 삶의 질은 43.9점으로 모두 일반 인구 평균보다 낮게 나타났다. 이는 선행연구와 비교할 때 NTM-PD 환자들의 삶의 질이 대체로 저하되어 있다는 점에서는 일치하나, 영역별 수준에는 차이가 있었다. 일본에서 NTM-PD 환자를 대상으로 동일한 도구로 측정한 Yamane 등[11]의 연구에서는 신체적 삶의 질이 평균 42.8점으로 정신적 삶의 질 47.3점보다 낮았는데, 본 연구 대상자는 신체적 삶의 질 점수보다 정신적 삶의 질이 더 낮은 양상을 보였다. 이러한 차이는 본 연구 대상자의 질병 진행 정도가 비교적 경미한 데 기인한 것으로 판단된다. 항산균 도말검사는 임상적, 방사선학적 질병 진행과 관련이 있는데[38], 선행연구에서 항산균 도말검사 양성 환자 비율은 81.8%이었으나[11] 본 연구에서는 31.5%로 본 연구 대상자의 질환 정도가 경미하여, 그 결과 신체 기능을 더 잘 유지할 수 있어 신체적 삶의 질 점수가 더 높게 나타난 것으로 해석된다. 반면 본 연구에서 정신적 삶의 질 점수가 신체적 영역보다 낮게 나타났다. 이러한 차이는 연구 시점과 대상자 특성의 차이가 영향을 미쳤을 가능성이 있다. Yamane 등[11]의 연구는 2016년∼2019년에 시행된 반면, 본 연구는 coronavirus disease 2019 팬데믹 이후에 수행되어 만성 호흡기 질환자들이 경험하는 사회적 제약과 감염 불안이 정신적 삶의 질에 부정적 영향을 미쳤을 가능성이 있다. 다만 본 연구에서는 팬데믹의 영향을 직접적으로 측정하지 않았으므로 이러한 해석에는 신중함이 필요하며, 추후 연구를 통해 보다 명확한 인과관계를 규명할 필요가 있다.

본 연구 대상자의 삶의 질 수준을 폐결핵 환자와 비교해 보면[12], NTM-PD 환자들의 삶의 질은 유사한 수준으로 낮았으나 그 양상에는 차이가 있다. 폐결핵 환자는 6개월의 비교적 짧은 치료 기간과 높은 완치율로 치료 과정에서 삶의 질이 회복되는 궤적을 보이는 반면[30], NTM-PD 환자는 약 18개월간의 장기 치료가 필요하며 균 음전율이 60%에 불과하고 재발률이 30%에 달한다[5,6]. 더욱이 NTM-PD는 전염성이 없음에도 불구하고 폐결핵과 유사한 질환으로 인식되어 사회적 오해를 받으며, 질병에 대한 사회적 인식 부족으로 인해 적절한 이해와 지지를 받기 어려운 상황이다. 이러한 질병의 만성적 특성과 치료 불확실성, 사회적 편견이 복합적으로 작용하여 신체적 증상이 경미함에도 불구하고 심리적 디스트레스가 심화되어 삶의 질이 저하된 것으로 해석된다. 따라서 전염성에 대한 사회적 오해 해소, 장기간 치료에 대한 심리적 지지, 그리고 치료 불확실성에 대한 정서적 대처를 포함한 맞춤형 간호중재 접근이 필요하다.

본 연구 결과 대상자의 신체적 삶의 질은 남성 대상자가 여성 대상자보다 높았고, 기혼인 대상자가 미혼인 경우보다 높았으며, 동거가족이 있는 대상자가 없는 경우보다, 직업이 있는 대상자가 없는 경우보다 유의하게 높게 나타났다. 정신적 삶의 질 또한 기혼자, 동거가족 있는 경우, 취업 상태인 경우 점수가 더 높게 나타났다. 이는 배우자나 가족의 존재가 대상자에게 정서적 지지와 돌봄을 제공하여 스트레스를 완화하고 의욕적으로 만들어주어 정신적 삶의 질을 높이고[39], 경제활동 또는 직업을 지속하여 안정적인 수입을 유지하는 등 사회적 역할을 계속하는 것이 환자의 정신적 안녕에 보호 효과를 줄 수 있음을 시사한다[40]. 따라서 NTM-PD 환자의 치료 과정에 가족의 참여를 유도하여 지지체계를 강화하고 지역사회 자원과 연계하여 사회적 역할을 지속할 수 있도록 지원할 필요가 있다.

본 연구에서 CRP 수치가 0.5 mg/dL 이하이거나 ESR이 20 mm/hr 이하인 대상자는 높은 수치를 보이는 대상자에 비해 신체적 및 정신적 삶의 질이 유의하게 높은 것으로 나타났다. 선행연구에서도 CRP 수치가 NTM-PD 환자의 신체적, 정신적 삶의 질에 직접적인 영향을 미치는 요인으로 보고된 바 있어[10], 염증 지표가 NTM-PD 환자의 건강관련 삶의 질에 영향을 미치는 중요한 요인임을 시사한다. CRP, ESR과 같은 염증성 지표의 상승은 전신 염증 반응을 반영하며, 근육량 감소, 피로감 증가, 신체 기능 저하 등을 유발하여[41] 환자의 전반적인 신체적 안녕을 저해한다. 또한 선행연구에 따르면 염증 지표의 지속적인 상승은 우울증 발생과 밀접한 관련이 있으며[42], 염증으로 인한 신체적 기능 저하가 일상생활의 제약을 초래하여 환자의 우울, 불안 등을 심화시켜 정신적 삶의 질까지 악화시키는 것으로 해석된다. 더불어 입원 치료 경험이 없는 대상자, 과거 폐질환 병력이 없는 대상자, 그리고 BMI가 비만군에 해당하는 대상자가 다른 군에 비해 유의하게 높은 정신적 삶의 질 점수를 보였다. 입원 치료 경험의 부재는 질병의 중증도가 상대적으로 낮음을 의미하며, 입원으로 인한 심리적 스트레스를 경험하지 않았기 때문으로 해석된다. 과거 폐질환 병력이 없는 경우 기존 폐 손상 없이 NTM-PD 치료가 진행되어 치료 반응이 양호하고 예후에 대한 긍정적 기대감을 가질 수 있다. 또한 BMI가 비만군에 해당하는 대상자는 만성 소모성 질환인 NTM-PD로 인한 체중감소나 영양결핍의 위험이 상대적으로 낮아 신체적 저항력이 높고 치료 반응이 우수할 가능성이 있다. 이와 같이 상대적으로 양호한 건강상태는 심리적 안정감을 제공하고 질병 부담감을 감소시켜 정신적 삶의 질에 긍정적인 영향을 미친 것으로 사료된다. 이러한 결과는 NTM-PD 환자의 삶의 질 향상을 위해 염증반응 조절, 질병 진행 모니터링을 통한 입원 예방, 동반 폐질환 관리, 그리고 적절한 영양상태 유지가 중요함을 시사한다.

본 연구에서 NTM-PD 환자의 건강관련 삶의 질에 영향을 미치는 요인을 분석한 결과, 증상경험과 불확실성은 신체적 및 정신적 삶의 질 모두에 공통적으로 부정적 영향을 미쳤고, 정신적 삶의 질에는 건강증진행위와 동거가족 유무, BMI 또한 유의한 영향요인으로 나타났다. 먼저 증상경험은 본 연구에서 신체적 및 정신적 삶의 질에 모두 가장 강력한 부정적 영향을 미치는 변수로 확인되었다. 본 연구 결과 대상자의 증상경험에서 가장 빈도가 높은 증상은 피로, 기침, 객담, 수면장애, 식욕부진 순으로 나타났다(Supplementary Table 1). 이러한 증상들은 대상자의 일상 활동과 신체 기능뿐만 아니라 심리사회적 안녕에도 부정적인 영향을 미쳐 삶의 질 저하를 초래하는 것으로 해석된다. 선행연구에서도 NTM-PD 환자에서 심한 증상 경험이 삶의 질을 현저히 저하시킨다는 점이 보고된 바 있으며[36], 특히 만성 객담은 사회적 위축 및 우울감을 유발하여 정신적 삶의 질을 악화시킨다[11]. 따라서 환자의 증상경험을 완화하기 위한 약물중재와 더불어, 호흡재활 프로그램, 기침 조절 교육, 기도 분비물 관리 및 에너지 보존 전략 등의 체계적이고 개별화된 중재 전략이 필요하다.

질병에 대한 불확실성은 신체적 삶의 질과 정신적 삶의 질에 모두 유의한 부정적 영향을 미치는 것으로 확인되었으며, 특히 정신적 영역에 더 큰 영향력을 보였다. 이는 질병의 예측 불가능성과 통제 불가능성으로 인해 환자가 경험하는 스트레스가 신체적 증상 악화로 이어져[43] 신체적 삶의 질에 영향을 미칠 수 있음을 의미한다. 또한 NTM-PD는 장기적 치료와 불확실한 예후로 인해 환자들에게 상당한 심리적 스트레스를 초래하며[7], 이는 치료과정에서의 자기효능감과 치료 순응을 저하시켜 삶의 질 저하로 이어질 수 있다[12]. 이에 불확실성을 관리하기 위한 간호 전략으로 명확한 정보 제공 및 치료 경과에 대한 정기적 상담과 심리적 지지 프로그램이 도움이 될 수 있으며, 특히 간호사는 환자와 의료진 간에 신뢰관계를 형성하고 환자가 질병에 대한 통제감을 유지할 수 있도록 지속적인 교육 및 상담을 제공해야 한다[18].

본 연구에서 대상자의 정신적 삶의 질에만 유의한 영향을 미친 요인을 살펴보면, 먼저 건강증진행위는 정신적 삶의 질에 긍정적 영향을 미치는 요인으로 나타났다. NTM-PD 환자를 대상으로 한 선행연구에서 운동 및 호흡관리, 영양교육 등을 받은 후 삶의 질, 특히 정신적 삶의 질이 향상된 결과를 보였다[44]. 이러한 개선 효과는 규칙적 운동으로 자아존중감이 향상되고 적절한 영양 관리가 피로감을 감소시켜 질병 관련 불안과 우울을 완화하기 때문으로 해석된다. 동기 강화, 목표설정, 긍정적 피드백 등의 동기 부여 전략들은 환자의 자기효능감을 높이는 데 중요한 역할을 하며, 이를 통해 건강증진행위를 촉진하는 촉매 작용을 한다[45]. 실제로 만성 폐질환 환자를 대상으로 한 연구에서 폐 재활을 활용한 중재가 운동 능력과 건강 관련 삶의 질을 향상시킨다는 결과가 보고되었으며[46], 이는 NTM-PD 환자에게도 유사한 긍정적 효과를 기대할 수 있음을 시사한다. 따라서 간호사는 환자 개인의 질병 상태와 치료 단계에 따른 맞춤형 교육 프로그램을 제공하고, 정기적인 상담을 통해 개별화된 동기 부여 전략을 적용하여 환자의 치료 순응도와 자기관리 능력을 향상시킴으로써 궁극적으로 삶의 질 개선에 기여할 수 있을 것이다. 또한 동거가족 유무가 정신적 삶의 질에 유의한 영향을 미친 것으로 나타났다. NTM-PD 환자와 유사한 폐결핵 환자를 대상으로 한 연구에서 가족의 지지를 중심으로 한 중재를 받은 대상자가 그렇지 않은 대상자보다 삶의 질, 특히 정신적 삶의 질을 개선하는데 효과적인 결과를 보여[39] 가족의 정서적 지지는 만성질환 환자의 심리적 안정과 질병 적응에 중요한 역할을 함을 알 수 있다. 따라서 간호사는 환자의 가족을 적극적으로 간호 과정에 참여시키고, 가족 지지가 부족한 환자에 대해서는 사회적 지지자원을 연계하여 정서적 지지체계를 구축할 필요가 있다. 마지막으로 본 연구에서는 BMI 비만군인 대상자가 다른 군에 비해 정신적 삶의 질 점수가 높게 나타났는데, BMI와 정신적 삶의 질과의 관계에 관한 선행연구는 일관된 결과를 보이지 않는다. 만성폐쇄성폐질환 환자를 대상으로 한 연구에서 질환이 경증에서 중등도인 환자에서는 BMI가 약 25 kg/m^2^인 환자가 삶의 질이 가장 좋았으나 전체적으로는 BMI와 삶의 질 간에는 비선형적 관계가 나타나 만성폐쇄성폐질환 환자를 질병 단계별로 계층화하여 최적의 BMI 관리가 필요하다고 하였다[47]. 그러나 우울증이 있는 만성폐쇄성폐질환 환자를 대상으로 한 연구에서는 과체중 환자 집단에서 BMI 감소가 불안 및 우울 증상을 감소시키고 삶의 질을 향상시키는 것으로 나타났다[48]. 따라서 BMI와 정신적 삶의 질 간의 관계를 명확하게 밝혀내기 위해서는 질적 연구를 통한 심층적 탐색과 전향적 추적조사를 통한 인과관계를 확인하는 연구가 필요하고, 임상적으로는 BMI만을 확인하는 것보다는 환자의 전반적인 영양 상태를 함께 평가하고 이를 바탕으로 적절한 식이 중재와 영양 상담을 제공해야 할 것이다.

본 연구는 기존 선행연구들이 질병의 임상적 특성이나 신체적 증상을 중심으로 삶의 질을 주로 다룬 한계를 극복하고, 신체적•정신적 건강상태뿐 아니라 질병 관련 심리사회적 요인과 개인의 건강관리행동까지 포함하는 다차원적인 영향요인을 분석하였다는 점에서 의의가 있다. 특히 환자의 신체적•정신적 삶의 질에 미치는 요인을 통합적으로 파악함으로써 전인적 간호 접근의 필요성을 강조하였으며, 구체적인 임상 실무 전략을 제시했다는 점에서 실질적인 기여가 있다. 그러나 본 연구는 다음과 같은 제한점을 가진다. 첫째, 횡단적 설계의 한계로 인과관계를 명확히 규명하기 어렵다. 둘째, 일부 임상적 지표(CRP, ESR)는 결측값이 많아 다중 대체법을 사용하였으나, 이로 인해 분석 결과의 변동성이 감소하였을 수 있으므로 해석에 주의가 필요하다. 셋째, 연구 대상자가 특정 지역 병원에서 모집된 한정적 집단이므로 일반화에 주의가 필요하다. 향후 연구에서는 이론 기반의 구조화된 연구 설계를 통해 변수 간 경로와 인과관계를 명확히 하고, 종단적 연구 설계 및 중재연구를 통해 본 연구에서 제시한 간호중재의 효과를 검증하고, 일반화 가능성을 높이기 위하여 다양한 지역과 의료기관에서 모집된 대상자를 활용하여 반복연구를 제언한다. 또한 본 연구에서 사용된 도구는 폐결핵, 진폐 환자 등을 대상으로 수정•보완한 도구로 NTM-PD 환자만의 증상의 심각성이나 빈도, 약물복용 이행, 면역관리 등의 항목이 부족하므로 이를 보완한 NTM-PD 환자 특이적 도구 개발 연구를 제언한다.

## 결론

본 연구는 NTM-PD 환자의 건강관련 삶의 질 수준을 확인하고, 이에 영향을 미치는 요인을 신체적•심리사회적 측면에서 분석하였다. 연구 결과, 증상경험과 불확실성은 신체적•정신적 삶의 질 모두에 공통적으로 부정적인 영향을 미쳤으며, 정신적 삶의 질은 이 외에도 건강증진행위, 동거가족 유무, 비만 수준 등의 영향을 받는 것으로 나타났다. 이는 삶의 질 향상을 위해 증상완화, 정보 제공 및 심리적 지지, 건강관리 행동 강화, 가족 참여 확대 등 통합적 접근이 필요함을 시사한다. 특히 장기간 치료와 불확실한 예후를 지닌 NTM-PD 환자의 특성을 고려할 때, 간호사는 대상자의 증상과 심리 상태를 지속적으로 모니터링하고, 전인적 간호중재를 통해 신체적•정신적 안녕을 향상시킬 수 있는 실무 전략을 설계해야 할 것이다. 향후에는 본 연구에서 제시된 영향요인을 기반으로 한 표준화된 간호중재 프로그램을 개발하고, 그 효과를 검증하는 중재연구 및 반복연구가 필요하다. 또한 다양한 지역과 진료기관에서 모집한 환자를 대상으로 일반화 가능성을 높이기 위한 후속 연구가 수행되어야 할 것이다.

## Figures and Tables

**Table 1.** Differences in Health-related Quality of Life According to Participants’ Characteristics (N = 130)

Characteristics	Categories	n (%) or M ± SD	HRQoL			
			PCS		MCS	
			M ± SD	t or F (p)	M ± SD	t or F (p)
Sex	Men	33 (25.4)	48.90 ± 5.84	2.32 (.024)	47.06 ± 12.07	1.75 (.086)
	Women	97 (74.6)	46.11 ± 6.36		42.89 ± 11.06	
Age (years)		65.56 ± 8.97				
	< 65	59 (45.4)	47.98 ± 6.54	1.91 (.058)	44.58 ± 12.29	0.57 (.573)
	≥ 65	71 (54.6)	45.85 ± 6.02		43.42 ± 10.71	
Marital status	Married	101 (77.7)	47.44 ± 6.18	2.08 (.044)	45.04 ± 11.07	1.97 (.041)
	Single	29 (22.3)	44.64 ± 6.47		40.14 ± 12.00	
Cohabiting family	Yes	109 (83.8)	47.34 ± 6.20	2.13 (.042)	44.96 ± 11.29	2.39 (.023)
	No	21 (16.2)	44.10 ± 6.43		38.70 ± 10.91	
Education	≤ Middle school	33 (25.4)	45.22 ± 5.86	2.03 (.135)	43.52 ± 10.90	2.39 (.096)
	≤ High school	54 (41.5)	46.73 ± 6.15		41.87 ± 11.45	
	≥ College	43 (33.1)	48.15 ± 6.74		46.88 ± 11.42	
Employed	Yes	48 (36.9)	48.69 ± 6.24	2.64 (.010)	46.58 ± 11.56	2.02 (.046)
	No	82 (63.1)	45.72 ± 6.16		42.40± 11.12	
Smoking status	Never smoker	103 (79.2)	46.87 ± 6.63	0.39 (.675)	43.85 ± 11.26	1.24 (.294)
	Ex-smoker	25 (19.3)	46.89 ± 5.16		45.29 ± 11.83	
	Active smoker	2 (1.5)	42.85 ± 3.51		32.22 ± 14.55	
NTM species	MAC	80 (61.5)	46.55 ± 5.82	0.35 (.789)	43.94 ± 10.49	1.03 (.382)
	*M. abscessus*	15 (11.6)	46.93 ± 7.83		39.66 ± 13.54	
	*M. massiliense*	12 (9.2)	48.57 ± 8.32		46.29 ± 11.71	
	Others	23 (17.7)	46.74 ± 6.14		45.57 ± 12.91	
Sputum smear	Positive	41 (31.5)	46.16 ± 3.73	−0.78 (.440)	43.03 ± 10.47	−0.65 (.518)
	Negative	89 (68.5)	47.12 ± 6.15		44.37 ± 11.87	
Cavitary lesion	Yes	34 (26.2)	46.83 ± 6.79	0.01 (.989)	41.75 ± 12.42	−1.24 (.221)
	No	96 (73.8)	46.81 ± 6.20		44.73 ± 11.01	
CRP (mg/dL) (n = 84)	≤ 0.5	67 (79.8)	47.98 ± 6.26	4.51 (<.001)	46.13 ± 11.99	3.50 (.002)
	> 0.5	17 (20.2)	40.67 ± 5.90		36.04 ± 10.23	
ESR (mm/hr) (n = 91)	≤ 20	44 (48.4)	48.83 ± 6.40	3.17 (.002)	46.37 ± 11.39	2.10 (.038)
	> 20	47 (51.6)	44.52 ± 6.59		41.14 ± 12.32	
Disease duration (months)		42.61 ± 27.7				
	≤ 12	18 (13.8)	46.87 ± 7.44	0.24 (.915)	41.17 ± 12.03	0.46 (.768)
	13∼24	22 (17.0)	47.15 ± 6.46		44.16 ± 12.08	
	25∼36	21 (16.2)	45.74 ± 6.90		43.79 ± 9.90	
	37∼60	38 (29.2)	47.36 ± 5.81		43.75 ± 13.59	
	≥ 61	31 (23.8)	46.61 ± 6.08		45.75 ± 8.70	
Treatment status	Never	14 (10.8)	46.03 ± 5.87	0.47 (.623)	43.02 ± 8.92	1.00 (.372)
	Previously	51 (39.2)	47.47 ± 5.82		45.71 ± 11.09	
	Currently	65 (50.0)	46.47 ± 6.84		42.77 ± 12.11	
History of hospitalization	Yes	49 (37.7)	45.37 ± 6.57	−2.01 (.048)	40.96 ± 11.56	−2.33 (.022)
	No	81 (62.3)	47.69 ± 6.05		45.75 ± 11.02	
Past lung disease	Yes	26 (20.0)	45.18 ± 6.00	−1.54 (.132)	39.03 ± 13.29	−2.19 (.036)
	No	104 (80.0)	47.23 ± 6.37		45.18 ± 10.62	
BMI	Underweight	36 (27.7)	44.94 ± 5.96	1.78 (.154)	41.58 ± 11.33	3.40 (.020)
	Normal weight	78 (60.0)	47.51 ± 6.34		43.40 ± 10.86	
	Overweight	9 (6.9)	48.95 ± 7.12		51.40 ± 10.27	
	Obese	7 (5.4)	45.98 ± 6.03		52.69 ± 13.86	

M = Mean; SD = Standard deviation; HRQoL = Health-related quality of life; PCS = Physical component summary; MCS = Mental component summary; NTM = Non-tuberculous mycobacteria; MAC = *Mycobacterium avium* complex; M. = Mycobacterium, CRP = C-reactive protein; ESR = Erythrocyte sedimentation rate; BMI = Body mass index.

**Table 2.** Levels of Symptom Experience, Illness Uncertainty, Health Promotion Behavior, and Health-related Quality of Life (N = 130)

Variables	Total M ± SD	Item M ± SD
Symptom experience	25.91 ± 4.92	1.62 ± 0.31
Illness uncertainty	93.96 ± 18.08	2.85 ± 0.55
Health promotion behavior	142.87 ± 20.08	3.57 ± 0.50
HRQoL		
PCS	46.82 ± 6.33	-
MCS	43.95 ± 11.42	-

M = Mean; SD = Standard deviation; HRQoL = Health-related quality of life; PCS = Physical component summary; MCS = Mental component summary.

**Table 3.** Correlations between Symptom Experience, Illness Uncertainty, Health Promotion Behavior, and Health-related Quality of Life (N = 130)

Variables	Illness uncertainty	Health promotion behavior	Health-related quality of life	
			PCS	MCS
	r (*p*)			
Symptom experience	.30 (< .001)	−.45 (< .001)	−.57 (< .001)	−.61 (< .001)
Illness uncertainty	1	−.47 (< .001)	−.43 (< .001)	−.48 (< .001)
Health promotion behavior	-	1	.39 (< .001)	.56 (< .001)

PCS = Physical component summary; MCS = Mental component summary.

**Table 4.** Factors Influencing Physical Component Summary of Health-related Quality of Life (N = 130)

Variables	B	SE	t	* p*
(Constant)	62.64	6.88	9.10	< .001
Sex (ref. women)	1.47	1.07	1.37	.170
Married (ref. single)	−0.45	1.37	−0.33	.742
Cohabiting family (ref. no)	2.45	1.53	1.60	.109
Job (ref. no)	1.09	0.96	1.13	.257
CRP (mg/dL) (ref. ≤0.5)	−2.23	1.53	−1.46	.155
ESR (mm/hr) (ref. ≤20)	−0.94	1.17	−0.80	.425
History of hospitalization (ref. no)	−0.25	1.00	−0.25	.802
Symptom experience	−0.48	0.11	−4.46	< .001
Illness uncertainty	−0.08	0.03	−2.85	.004
Health promotion behavior	0.02	0.03	0.69	.492
F = 9.66∼10.90 (*p *< .001), R^2 ^= .45∼.58, Adj. R^2 ^= .40∼.53				

SE = Standard error; ref. = Reference; CRP = C-reactive protein; ESR = Erythrocyte sedimentation rate.

**Table 5.** Factors Influencing Mental Component Summary of Health-related Quality of Life (N = 130)

Variables	B	SE	t	* p*
(Constant)	56.92	10.64	5.35	< .001
Married (ref. single)	−1.37	2.21	−0.62	.534
Cohabiting family (ref. no)	4.93	2.46	2.01	.045
Job (ref. no)	0.04	1.51	0.03	.977
CRP (mg/dL) (ref. ≤0.5)	−1.38	2.21	−0.62	.534
ESR (mm/hr) (ref. ≤20)	−1.19	1.78	−0.67	.507
History of hospitalization (ref. no)	−1.06	1.57	−0.67	.500
BMI (ref. normal weight)				
Underweight	1.34	1.71	0.79	.431
Overweight	1.93	2.98	0.65	.516
Obese	9.24	3.17	2.92	.004
Symptom experience	−0.89	0.17	−5.24	< .001
Illness uncertainty	−0.15	0.05	−3.26	.001
Health promotion behavior	0.15	0.04	3.41	.001
F = 8.80∼13.93 (*p *< .001), R^2 ^= .58∼.61, Adj. R^2 ^= .53∼.55				

SE = Standard error; ref. = Reference; CRP = C-reactive protein; ESR = Erythrocyte sedimentation rate; BMI = Body mass index.

## Data Availability

Please contact the corresponding author for data availability.

## Supplementary Material

Supplementary Table 1. Severity Level of Symptom Experience
