# Supplementary material for: Factors influencing health-related quality of life among patients with non-tuberculous mycobacterial pulmonary disease in South Korea: a cross-sectional survey study
Source: J Korean Biol Nurs Sci. 2025 Nov 21;27(4):671–83. [Article in Korean] doi: 10.7586/jkbns.25.042 (PMC13268283; doi:10.7586/jkbns.25.042)
Supplement: Supplementary file 1 [file jkbns-25-042-Supplementary-Table-1.pdf]

Supplementary Table 1. Severity Level of Symptom Experience (N = 130)

Variables	Item M ± SD
1. Cough	2.03 ± 0.71
2. Sputum	1.94 ± 0.68
3. Dyspnea	1.48 ± 0.68
4. Fatigue	2.10 ± 0.83
5. Loss of appetite (anorexia or decreased appetite, etc.)	1.69 ± 0.72
6. Fever	1.28 ± 0.50
7. Pain (chest pain, headache, generalized pain, etc.)	1.68 ± 0.78
8. Gastrointestinal disturbance (nausea, vomiting, etc.)	1.58 ± 0.65
9. Skin abnormality (skin heat sensation, skin rash, etc.)	1.52 ± 0.66
10. Hemoptysis	1.31 ± 0.54
11. Cold sweats	1.47 ± 0.55
12. Peripheral neuropathy (numbness in hands and feet)	1.61 ± 0.70
13. Hearing impairment (hearing loss, tinnitus, vertigo)	1.49 ± 0.76
14. Visual disturbance (blurred vision, decreased visual acuity)	1.64 ± 0.67
15. Bowel and bladder dysfunction	1.38 ± 0.59
16. Sleep disturbance	1.72 ± 0.81

M = Mean; SD = Standard deviation.
